# Alterations in the Poison Schedule under the Poisons and Pharmacy Act

**Published:** 1921-01-08

**Authors:** 


### Alterations in the Poison Schedule under the Poisons and Pharmacy Act.

New regulations governing the selling of poisonous substances came into force on January 1, 1921. The most important changes are that coca, its preparations and mixtures containing 0.1 per cent. of alkaloids is now substituted for 1 per cent. of alkaloids. Heroin and all preparations containing 0.1 per cent. or more of the drug is added to *Part 1* of the schedule; also ecgonine and all preparations of the drug which contain 0.1 per cent. or more. Opium and all preparations containing 0.2 per cent. or more of morphine is substituted for 0.75 per cent., as in the former schedule. In Part 2 of the schedule, zinc chloride is added, except that preparations used for soldering and industrial processes are exempt, provided that they are sold in closed vessels and labelled "poisonous," with name and address of seller.

It may be explained that substances in schedule (Part 1) can only be supplied by a pharmacist to a person known to him, and the sale must be recorded in a special poison register and signed by the purchaser. This safeguard, whilst it may not altogether obviate poisoning either accidentally or by intent, has proved of great value on several occasions, notably in the "Crippen" case, in the detection of crime by poisoning. Substances in Part 2 of the schedule can be supplied without obtaining a signature, provided that the container is labelled "poisonous" and the name and address of the seller also appears on the label.

